# Family intimacy and adaptability and non-suicidal self-injury: a mediation analysis

**DOI:** 10.1186/s12888-024-05642-1

**Published:** 2024-03-18

**Authors:** Yuehong Gao, Yanchi Wang, Zhiping Wang, Mingzhen Ma, Hongjiao Li, Jinhong Wang, Jianan Liu, Huaying Qian, Ping Zhu, Xujuan Xu

**Affiliations:** 1 The Fourth People’s Hospital of Nantong, Nantong, China; 2 The Sixth People’s Hospital of Nantong, Nantong, China; 3 Nantong University, Nantong, China; 4 Nanjing Brain Hospital, Nanjing, China; 5 Suzhou Guangji Hospital, Su Zhou Shi, China; 6 Affiliated Hospital of Nantong University, Nantong, China

**Keywords:** Adolescent depression, Family intimacy and adaptability, Psychological resilience, Depression severity, Non-suicidal self-injury

## Abstract

**Background:**

Current research has been focusing on non-suicidal self-injury (NSSI) behaviors among adolescents with depression. Although family intimacy and adaptability are considered protective factors for NSSI, evidence supporting this relationship is lacking.

**Objective:**

This study aims to examine the mechanisms operating in the relationship between family intimacy and adaptability and NSSI behaviors among adolescents.

**Methods:**

A self-administered general demographic information questionnaire, the Behavioral Functional Assessment Scale for Non-Suicidal Self-Injury, the Family Intimacy and Adaptability Scale, the Connor-Davidson Resilience Scale, and the Self-Assessment of Depression Scale were distributed among adolescents with depression in three tertiary hospitals in Jiangsu Province.

**Results:**

The relationship between family intimacy and adaptability and NSSI was assessed among 596 adolescents with depression. The results revealed the following: (1) Family intimacy and adaptability were negatively correlated with NSSI behavior. (2) Psychological resilience and depression levels acted as chain mediators in the relationship between family intimacy and adaptability and NSSI behavior.

**Conclusions:**

Enhancing psychological resilience, controlling depressive symptoms, and reducing depression severity among adolescents by improving their family intimacy and adaptability are conducive to preventing and mitigating their NSSI behaviors.

## Introduction

Non-suicidal self-injury (NSSI) refers to the direct, intentional, and repeated destruction of bodily tissues without suicidal intent, including wrist cutting, skin scratching, biting, pinching, and head banging [1]. Although NSSI is socially unacceptable, in recent years, its incidence among adolescents has been steadily increasing and become a major public health concern that seriously threatens their physical and mental health worldwide [2]. Adolescence is a critical period of physical and mental development. Cognitive development, hormonal changes, and life events (e.g., tremendous academic pressure) not only affect adolescents’ emotional disposition but also increase their risk of developing NSSI behaviors [3]. Notably, NSSI behaviors are believed to habituate individuals to pain, thus increasing their tolerance—while reducing their fear—of such behaviors [4, 5]. Reportedly, individuals who have acquired the capability for suicide through NSSI engagement are less afraid of what a lethal suicide attempt entails and are more likely to mentally rehearse suicide plans, further reducing their fear of death [6]. More than 70% of adolescents exhibiting NSSI experience suicidal thoughts [7].

The family—as an important place for individual physical and mental development—plays a vital role in developing adolescents’ mental health; relevant studies have indicated that effective family functioning reduces adolescents’ depressive symptoms and self-injury behavior [8]. Among adolescents, family environment dysfunction—including family conflict, parent–child relationship disharmony, and an inappropriate expression of parental emotions—influences the adoption of poor emotion regulation strategies, which, in turn, influences NSSI behaviors [9]. According to Olson et al. [10], the primary components of family functioning are family intimacy and adaptability. Family intimacy refers to the emotional bond among family members, while family adaptability refers to the family system’s ability to respond to situational and developmental stress. Family intimacy and adaptability—reflecting an individual’s perceived emotional connection to the family—are indicators of the emotional closeness of family relationships and positive family climate.

One study found that individuals from dysfunctional familys cannot rationally regulate their emotions and tend to resort to extreme methods, such as self-injury, when experiencing negative emotions that they cannot cope with [11]. Teenagers exhibiting—compared with those not exhibiting—self-injury often have families with evident functional obstacles, including confusion, inadequate communication, and overprotection [12]. On the contrary, a warm, responsive, and supportive family reduces the frequency of negative thoughts and maladaptive behaviors among teenagers [13, 14]. Prior research has demonstrated the effects of family intimacy and adaptability on NSSI behaviors among adolescents. Accordingly, hypothesis H1 is proposed as follows: Family intimacy and adaptability influence NSSI behaviors among adolescents.

Psychological resilience, also called mental resilience or mental toughness, refers to an individual’s ability to adapt positively to adversity. As an essential element of positive psychology, psychological resilience has become a hotspot of mental health research over the past 30 years [15]. Psychological resilience is a dynamic process that refers to individuals’ capacity to cope with stressors and difficulties while maintaining normal psychological and physical functioning [16]. Resilience reduces risk factors’ negative effects on individual development; that is, adverse events disrupt individuals’ original state of physical and mental equilibrium, and individuals optimally attempt to mobilize various protective factors to restore and maintain this equilibrium [17]. Adolescence is a significant developmental stage for psychological resilience. Through the experiential acquisition of skills to cope with adversity and these skills’ influence on internal factors, biological and sociopsychological factors are molded via epigenetic mechanisms, and finally, psychological resilience is produced [18].

One study found that family intimacy and adaptability are protective factors for psychological resilience and that parental support, caring relationships members reduce or moderate the negative risk outcomes, thus promoting positive outcomes [19]. Accordingly, hypothesis H2 is proposed as follows: Family intimacy and adaptability influence mental resilience among adolescents with depression.

Studies have indicated that resilience alleviates mental health problems and prevents NSSI behaviors [20, 21]. Reportedly, high psychological resilience reduces the likelihood of engaging in NSSI behaviors [22]. Zhang’s study found that psychological resilience exerts a protective effect on the development of suicidal ideation among patients with depression and that a high level of psychological resilience reduces the likelihood of suicidal ideation [23]. Accordingly, hypothesis H3 is proposed as follows: Psychological resilience among adolescents with depression influences NSSI behaviors.

Per the World Health Organization’s latest estimates, around 350 million individuals worldwide suffer from depression, with an average of 1 in 20 people having had or currently having a depressive disorder, which is clinically manifested by significant and persistent depressed mood, a loss of interest, a lack of pleasure, a loss of energy, and reduced activity [24]. When adolescents face undesirable life experiences, such as stress or family dysfunction, they may struggle to express their emotions appropriately, thereby resulting in emotional disorders, strong emotional reactions, and a lack of emotional expression. One clinical study found that adolescents with positive—compared with those with negative—family functioning responded better to depression treatment and exhibited more significant decreases in depression levels over time during treatment [25, 26]. Accordingly, hypothesis H4 is proposed as follows: Family intimacy and adaptability influence depression severity among adolescents with depression.

According to Zhang, the depression level is an influential factor in NSSI incidence among adolescents [27]. The depression level is higher among adolescents with depression exhibiting—than among those not exhibiting—NSSI behaviors, suggesting that the higher the depression level, the higher the likelihood of NSSI [28]. Accordingly, hypothesis H5 is proposed as follows: Depression severity affects NSSI.

Along with exploring the mediating roles of psychological resilience and depression levels in family intimacy and adaptability with NSSI behaviors, this study focused on the association between psychological resilience and depression levels [29]. Studies have reported that individuals with higher—than those with lower—psychological resilience levels have a more positive cognitive thinking style and higher levels of self-acceptance in difficult situations, and can use internal and external protective factors to cope with difficult situations more promptly, thus exhibiting better adaptive capacity and lower susceptibility to depression [30].

Recently, the relationship between mental resilience and depression has attracted increasing attention. Mental resilience—as an important element of positive psychology—has gradually become a hotspot of mental health research in the past 30 years [31]. Zhu reported that psychological resilience weakens negative life events’ adverse impact on depression and protects individuals against this impact [32]. Li found that satisfactory mental resilience reduces the risk of depression among adults who have experienced childhood trauma [33]. One study found that psychological resilience improves an individual’s negative emotions when experiencing adversity and can be utilized as an internal defense mechanism to regulate suicidal ideation and depression [34]. Psychological resilience can help individuals cope with depressive symptoms, anxiety, and various negative emotions [35]. Accordingly, hypothesis H6 is proposed as follows: Psychological resilience influences depression severity.

## Materials and methods

### Participants

In this study, 612 adolescents diagnosed with and treated for depression at Nantong Fourth People’s Hospital, Nanjing Brain Hospital, and Suzhou Guangji Hospital between December 2022 and July 2023 were included. Of these, 16 were excluded owing to missing data (seven did not complete all the scales, and nine had several unanswered questions). Finally, 596 adolescents were included in the analysis. The inclusion criteria were as follows: adolescents 1) aged 11–18 years; 2) [36] assigned ICD-10 diagnostic codes for depression F32.0, F32.1, and F32.2: Essentials of Clinical Description and Diagnosis [37] (i.e., persistent depressed mood or decreased interest in activities for at least two weeks, accompanied by difficulty concentrating, feelings of worthlessness, excessive and inappropriate feelings of guilt and self-blame, and hopelessness); 3) who agreed to participate in the study voluntarily or whose legal guardian consented to their participation. The exclusion criteria were as follows: adolescents with 1) mental disorders other than depression, 2) a history of alcohol or drug abuse, 3) serious physical illness, 4) abnormal hearing or vision, 5) any suicidal intention or behaviors(e.g., jumping from heights, hanging oneself, consuming a lethal dose of drugs) during the period of self-injury, and 6) refusal to participate in the study.

### Procedure

#### Sample size calculation

The sample size was calculated using an online sample size calculation software, namely, Monte Carlo Power Analysis for Indirect Effects. Model 6 is selected to calculate the sample size. A minimum sample size of 460 cases was considered sufficient to achieve statistical power, and 596 cases were selected for inclusion in the final analysis.

#### Data collection

Before commencing data collection, participants and their family/legal guardians were explained the study’s purpose, content, and significance. After obtaining informed consent, data collection was performed by professionally trained personnel. Data were collected at the end of consultations for outpatients and after stabilization for inpatients in quiet, comfortable, well-lit, and private locations to avoid external factors’ interference. The researchers employed a set of standardized instructions to aid participants in completing the questionnaire; when participants encountered uncertainty, the researchers provided guidance in a consistent and timely manner.

### Ethical considerations

The following principles guided this study: First, the informed consent principle was followed; all participants (or their parents/legal guardians) were presented with a standardized “Informed Consent Form,” which outlined the study’s purpose, content, and significance. Second, the autonomy principle was followed by ensuring voluntary participation. Third, the confidentiality principle was followed; the researchers used codes to ensure the security of participants’ data. Additionally, the participants names were not published to prevent any possible leakage of information. Fourth, the benefit and non-harm principle was followed. This study was approved by the Ethics Committee of the Fourth People’s Hospital of Nantong City (approval no.: 2022-Ko37).

### Statistical analysis

Data were analyzed using SPSS 25.0 software. Two independent samples t-tests were employed for continuous variables (i.e., age) that followed a normal distribution to compare the NSSI and non-NSSI groups. Categorical data (i.e., gender, residence, delivery mode, and whether an only child) were described statistically using percentages; the chi-squared test was employed to assess whether a difference existed in NSSI incidence among adolescents with depression. Pearson correlation analyses were performed for family intimacy and adaptability, psychological resilience, depression, and NSSI. Notably, SPSS PROCESS Component Model 6 was employed to assess the mediating role of psychological resilience and depression level in the relationship between family intimacy and adaptability and NSSI; age, gender, and place of residence were controlled for. The bootstrap method—with a test level of α = 0.05—was used to examine the mediating role of psychological resilience and depression level.

## Results

The final analysis included 596 adolescents with depression, including 212 (35.57%) boys and 384 (64.43%) girls. Of these, 364 (61.07%) had experienced NSSI, whereas 232 (38.93%) had not. Data regarding age, gender, and place of residence were obtained. No significant differences were observed in terms of age and place of residence between the NSSI and non-NSSI groups (*P* > 0.05). However, a statistically significant difference was observed in terms of gender between both groups (*P* < 0.05; Table 1).

**Table 1:** The general situation of the study object

Item	NSSI	test	statistic	*P*
	Yes (*n* = 364)	No (*n* = 232)		
Sex n(%)				
Male	115 (54.2)	97 (37.7)	6.454^b^	0.014
Female	249 (64.8)	135 (35.2)		
Residence n(%)				
city	184 (60.5)	120 (39.5)	0.078^b^	0.780
village	180 (61.6)	112 (38.4)		
Age m ± sd	14.96 ± 1.841	14.96 ± 1.908	-1.020^a^	0.984

^a^t

^b^x^2^

### Correlations between family intimacy and adaptability, psychological resilience, depression severity, and NSSI

The mean scores of family intimacy and adaptability and psychological resilience in the NSSI group were lower than those in the non-NSSI group, and the difference was statistically significant (*P* < 0.001). The SDS’ mean scores in the NSSI group were higher than those in the non-NSSI group, and the difference was statistically significant (*P* < 0.001; Table 2).

**Table 2:** Comparison of family intimacy and adaptability, mental resilience and depression between NSSI group and non-NSSI group

	Item			NSSI	test statistic	*P*
			Yes (*n* = 364)	No (*n* = 232)		
FACES II	m ± sd	Family Intimacy	41.74 ± 10.13	45.20 ± 10.07	4.080	0.000
		Family Adaptability	31.83 ± 10.17	37.05 ± 9.93	6.168	0.000
CD-RISC	m ± sd		58.74 ± 18.077	64.76 ± 15.952	4.147	0.000
SDS	m ± sd		68.37 ± 11.608	63.24 ± 9.640	-5.130	0.000

Family intimacy positively impacted psychological resilience (R2 = 0.371, F = 5.6904); the higher the family intimacy scores, the higher the psychological resilience scores. Family intimacy and psychological resilience exerted an inhibitory effect on the depression level (R2 = 0.0610, F = 7.6547); the higher the family intimacy and psychological resilience scores, the lower the depression level scores. Family intimacy and psychological resilience exerted an inhibitory effect on NSSI, whereas depression level exerted a facilitatory effect on NSSI; the higher the family intimacy and psychological resilience scores, the lower the NSSI incidence (Table 3).

**Table 3:** Mean value, standard deviation, and correlation coefficient of Family intimacy, Psychological resilience and Degree of depression

Variable R		Family intimacy		Psychological resilience		Degree of depression	
		P	LLCT /ULCT	P	LLCT /ULCT	P	LLCT /ULCT
Psychological resilience	0.1685	0.000	0.15 /0.42				
Degree of depression	0.2033	0.000	-0.25/-0.08	0.000	-0.12/-0.02		
Non-suicidal self-injury	0.1732	0.007	-0.04/-0.00	0.002	-0.03 /-0.01	0.000	0.02 /0.05
M		43.06			61.09		66.38
SD		10.24			17.52		11.16

*LLCT* Lower bound of 95% confidence interval, *ULCT* Upper 95% confidence interval

Family adaptability positively impacted psychological resilience (R2 = 0.0298, F = 18.2077); the higher the family adaptability scores, the higher the psychological resilience scores. Family adaptability and psychological resilience exerted an inhibitory effect on the depression level (R2 = 0.0548, F = 17.1740); the higher the family adaptability and psychological resilience scores, the lower the depression level scores. Family adaptability and psychological resilience exerted an inhibitory effect on NSSI, whereas the depression level exerted a facilitatory effect on NSSI; the higher the family adaptability and psychological resilience scores, the lower the NSSI incidence (Table 4).

**Table 4:** Mean value, standard deviation, and correlation coefficient of Family adaptability, Psychological resilience and Degree of depression

Variable	R	Family adaptability		Psychological resilience		Degree of depression	
		P	LLCT /ULCT	P	LLCT /ULCT	P	LLCT /ULCT
Psychological resilience	0.1726	0.000	0.16 /0.43				
Degree of depression	0.2342	0.000	-0.29/-0.12	0.000	-0.12/-0.02		
Non-suicidal self-injury	0.1432	0.000	-0.06/-0.02	0.002	-0.03 /-0.00	0.000	0.02 /0.05
M		33.86			61.09		66.38
SD		10.39			17.52		11.16

*LLCT* Lower bound of 95% confidence interval, *ULCT* Upper 95% confidence interval

### Mediating roles of psychological resilience and depression level in the relationship between family intimacy and adaptability and NSSI

**Fig. 1:**
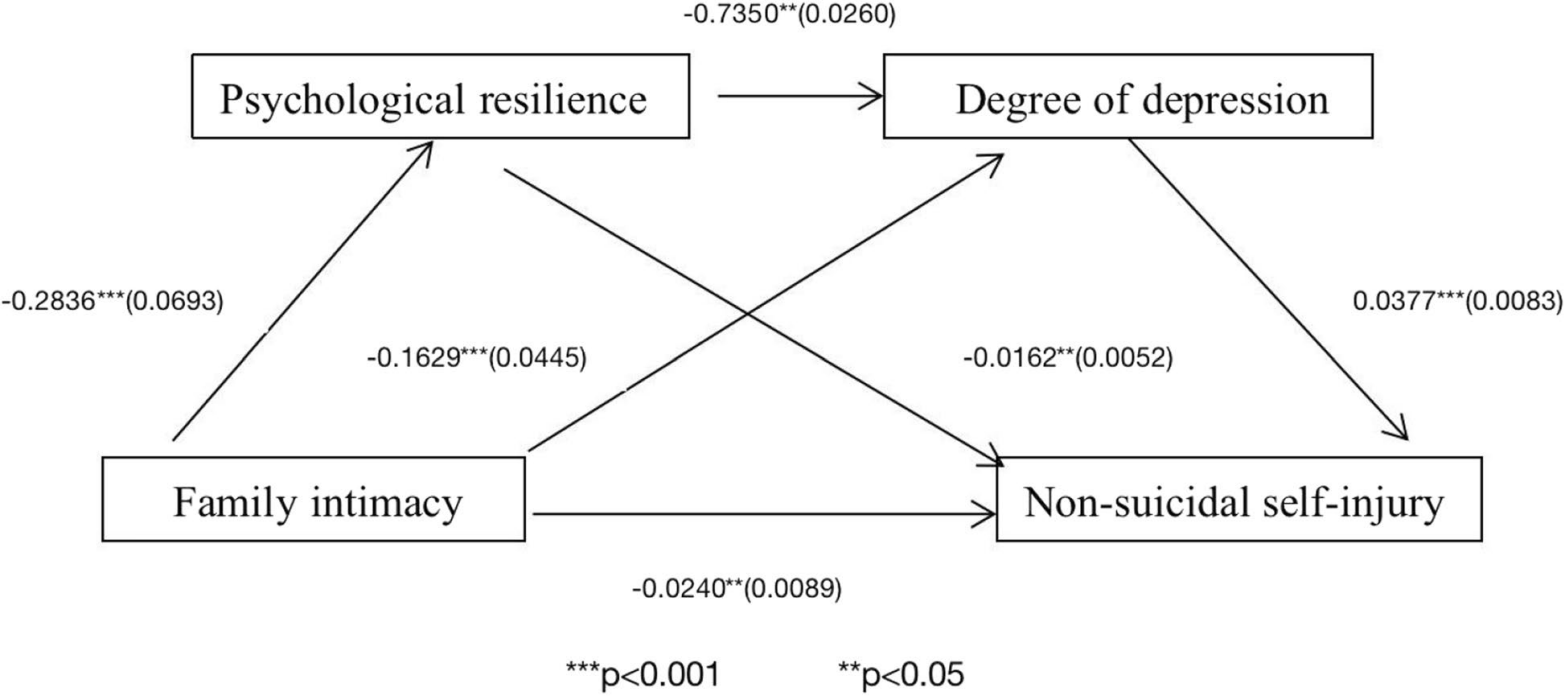
Mediation model of psychological resilience and depression level role family intimacy and NSSI

Notably, SPSS PROCESS Component Model 6 was utilized to evaluate the mediating roles of psychological resilience and depression level in the relationship between family intimacy and NSSI; age, gender, and place of residence were controlled for. Bootstrap techniques were employed to assess the mediating roles of psychological resilience, which exhibited an indirect effect of -0.0021 (95% confidence interval [CI] = [-0.0046, 0.0019]); depression level, which exhibited an indirect effect of -0.0029 (95% CI = [-0.0061,-0.0022]); and psychological resilience and depression level, which exhibited an indirect effect of -0.0003 (95% CI = [-0.0008,-0.0004]). None of the CIs passed through 0, indicating that the indirect effect holds. Family intimacy’s direct effect on NSSI was -0.0240 (95% CI = [0.0414,-0.0066]); none of the CIs passed through 0, indicating that the direct effect holds. The total effect was 0.0115 (Table 5; Fig. 1).

**Table 5:** Mediation effect analysis and its effect size (Family intimacy)

Effect	Path	Effect size	95% confidence interval	
Indirect effect	①- >④	-.0240	-0.04	-0.00
Intermediate effect	①- > ②-> ④ ①- > ③- > ④	-.0046 -.0061	-0.01 -0.01	-0.00 -0.00
	①- > ②-> ③- > ④	-.0008	-0.00	-0.00
Total Mediating Effect		-.0115	-0.02	-0.01

①Family intimacy; ②Psychological resilience; ③Degree of depression; ④Non-suicidal self-injury

**Fig. 2:**
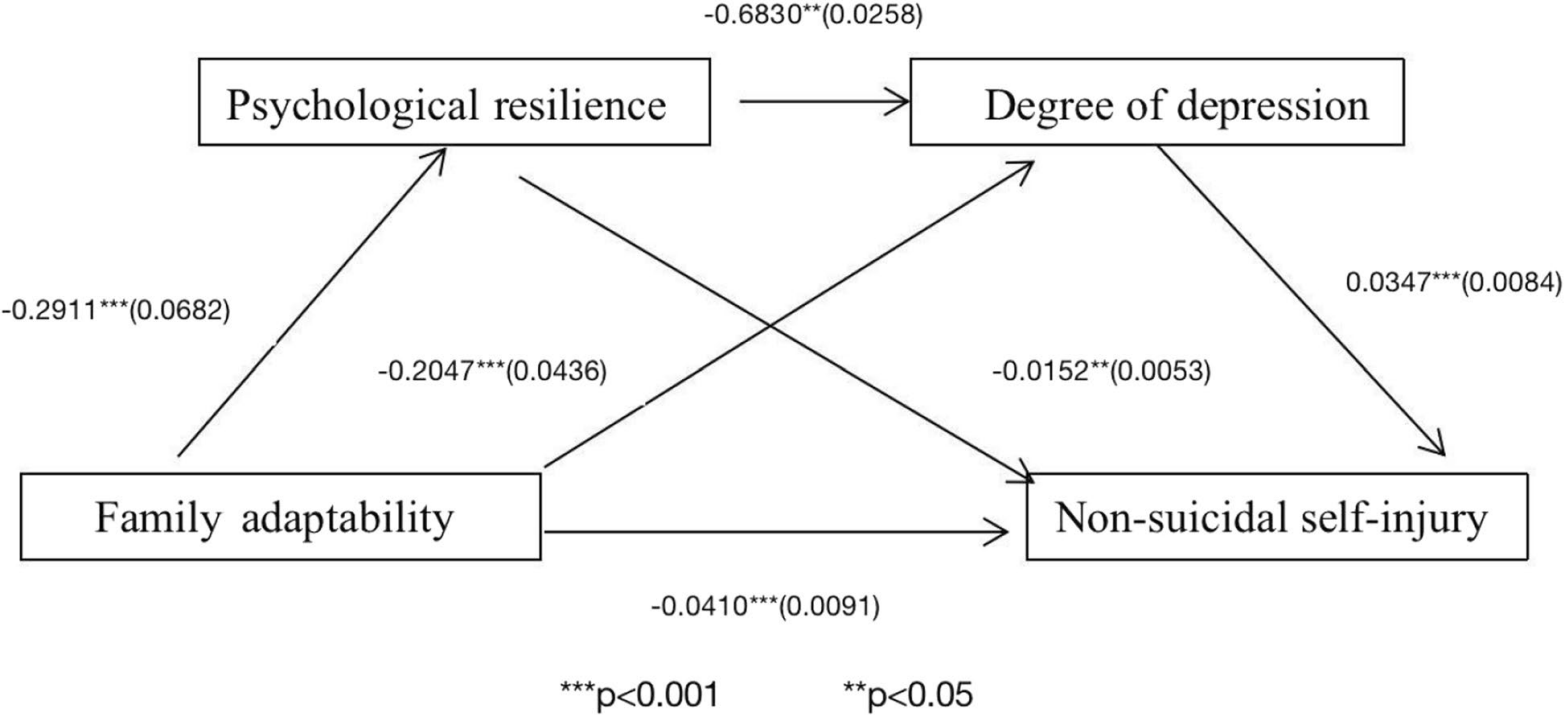
Mediation model of psychological resilience and depression level role family adaptability and NSSI

Further, the analysis revealed the mediating roles of psychological resilience, which exhibited an indirect effect of -0.0044 (95% confidence interval [CI] = [-0.0080, 0.0013]); depression level, which exhibited an indirect effect of -0.0071 (95% CI = [-0.0121,-0.0033]); and psychological resilience and depression level, which exhibited an indirect effect of -0.0071(95% CI = [-0.0016,-0.0001]); none of the CIs passed through 0, indicating that the indirect effect holds. Family adaptability’s direct effect on NSSI was -0.0410 (95% CI = [0.0588,-0.0233]); none of the CIs passed through 0, indicating that the direct effect holds. The total effect was 0.0122 (Table 6; Fig. 2).

**Table 6:** Mediation effect analysis and its effect size (Family adaptability)

Effect	Path	Effect size	95% confidence interval	
Indirect effect	①-> ④	-.0410	-0.06	-0.02
Intermediate effect	①- > ②->④ ①- > ③- > ④	-.0044 -.0071	-0.01 -0.01	-0.00 -0.00
	①- > ②-> ③- > ④	-.0007	-0.00	-0.00
Total Mediating effect		-.0122	-0.02	-0.01

①Family adaptability; ②Psychological resilience; ③Degree of depression; ④Non-suicidal self-injury

## Discussion

In this study, the NSSI incidence among adolescents with depression was 61.07%—higher than the 51% reported in previous studies [38]. This increase may be because some previous studies did not standardize the NSSI assessment scale’s use to determine NSSI incidence, whereas we used the Functional Assessment Scale for Non-Suicidal Self-Injury Behaviors in Adolescents—a scale that refines NSSI behaviors and broadly encompasses aspects that surpass what are traditionally considered as NSSI behaviors. Another reason may be that during the coronavirus disease 2019 pandemic, pre-existing mental health problems among adolescents may have worsened, thereby increasing NSSI incidence.

Hu’s study on family environment’s effect on self-injury during the pandemic further substantiates the above argument [39]. Consistent with previous findings, this study found a strong negative association between family intimacy and adaptability and NSSI behaviors [40–42]. Families with high intimacy create a favorable living environment for adolescents, which is crucial for their physical and mental development. Therefore, increasing family intimacy and improving parent–child relationships can serve as preventive measures and effective avenues for early intervention to reduce NSSI incidence among adolescents with depression [43].

Per our findings, psychological resilience plays a mediating role in the relationship between family intimacy and adaptability and NSSI behaviors—consistent with a previous study [14]. The psychological resilience of the NSSI group was 6 points lower than that of the non-NSSI group and 33 points lower than the national normative score for middle school students [44]. The correlational analyses revealed that self-injury behaviors negatively correlated with total family intimacy and adaptability and psychological resilience scores, suggesting that both family intimacy and adaptability and psychological resilience significantly influence self-injury behavior. This can be further explained by the model of adolescent psychological development [19], which explains self-injury behaviors’ internal mechanism through the combined effect of internal and external factors of “environmental competence-behavior,” whereby effective parenting and high mental competence levels motivate adolescents to engage in relatively positive behaviors. Family intimacy and adaptability—as crucial measures of family functioning and environment—can impact experiential avoidance through psychological resilience, which refers to an individual’s ability to protect themselves from hindered growth when experiencing adversity; the higher the psychological resilience level, the better the adaptive capacity [45], the more effective the self-regulation when facing difficulties and setbacks, and the more effective the self-regulation, thereby mitigating and buffering the negative impact on experiential avoidance precipitated by low family intimacy and adaptive status [46]. Therefore, hospitals and schools should focus on and fully utilize psychological resilience’s protective role and mechanism of psychological resilience in regard to adolescent in adolescents’ family functioning and mental health while also preventing adolescent self-injury behaviors by providing courses on emergency and crisis psychological intervention. Moreover, the theory of positive adolescent development suggests that activating adolescents’ positive environmental characteristics and enhancing their positive growth capacity are important factors in ensuring their healthy development [47].

Furthermore, depression severity was found to play a mediating role in the relationship between family intimacy and adaptability and NSSI behaviors—consistent with a previous study [42]. Per the mediation effect analyses, family environment directly as well as indirectly influences NSSI incidence among adolescents through depression level’s mediating role. The higher the dysfunction level in the family environment, the more likely adolescents are to develop depressive disorders; the higher the depression level, the more NSSI behaviors are likely to occur, consistent with a previous study’s findings [48]. Adolescents tend to relieve their negative emotions through NSSI behaviors, and a negative family environment is among the most important influencing factors for adolescents to experience depressive emotions [49]. Various problems pertaining to family environment dysfunction, including parent–child alienation, parental over-criticism, and harsh parental control, tend to precipitate psychological disorders among adolescents, which, in turn, is associated with NSSI [50].

Theoretically, existing studies on NSSI’s influence on adolescent depression have predominantly focused on the influencing factors. This study not only explored the direct effects of family intimacy and adaptability on NSSI but also examined the intrinsic influence mechanisms, thereby enriching the literature on NSSI among adolescents with depression to a certain extent and providing novel ideas for preventing NSSI incidence. Per this study’s findings, family intimacy and adaptability and psychological resilience are all negatively associated with depression severity and NSSI. This study’s results provide crucial insights for hospitals and schools in preventing NSSI among adolescents.

This study has several limitations. This study adopted a cross-sectional design that could not assess the causal relationships among family intimacy and adaptability, psychological resilience, depression levels, and self-injury behaviors among adolescents with depression; future longitudinal studies are necessary to elucidate the causal and temporal relationships among the various variables. Second, numerous factors influence NSSI behaviors among adolescents with depression, and perhaps, other individual characteristics (e.g., self-esteem level) and environmental factors (e.g., social support) may exert an impact. Subsequent studies can further explore other factors that may affect NSSI, to improve further the research on NSSI’s mechanism among adolescents with depression. This study’s sample was derived from cities above the second class in China, and patients from rural areas in remote mountainous regions could not be included; hence, this study’s findings may be **s**omewhat regionally biased.

## Conclusion

Psychological resilience and depression level mediate the relationship between family intimacy and adaptability and NSSI among adolescents with depression. Hence, early intervention for depressive disorders is crucial to prevent and control NSSI incidence among adolescents. Increasing positive interactions among family members, strengthening parent–child relationships, and creating a healthy family environment can provide support for growing adolescents. Additionally, schools and families should focus on adolescents’ mental health, provide timely psychological interventions, and enhance their psychological resilience to help them resist negative experiences’ adverse effects and prevent the somatization of psychological problems.
